# An alternative technique for Descemet’s membrane detachment following phacoemulsification: case report and review of literature

**DOI:** 10.1186/s12886-017-0506-3

**Published:** 2017-06-29

**Authors:** Yan Weng, Yu-ping Ren, Li Zhang, Xiao-dan Huang, Xing-chao Shen-tu

**Affiliations:** 1 Eye Center, the Second Affiliated Hospital of Zhejiang University School of Medicine, Hangzhou, 310009 China; 2 Shaoxing Hospital of Traditional Chinese Medicine, Shaoxing, 312000 China

**Keywords:** Descemet’s membrane detachment, Phacoemulsification, Descemetopexy, Case report

## Abstract

**Background:**

Descemet’s membrane detachment (DMD) is one of the most serious complications of modern cataract surgery. We present an alternative technique for management of DMD with a review of the literature on current strategies for the treatment of DMD.

**Case presentation:**

A 74-year-old woman developed DMD after phacoemulsification and failed the first descemetopexy with air tamponade. An alternative method was used to drain the pre-descematic fluid and reposition the detached Descemet’s membrane in this rare case. This technique involved completely filling the anterior chamber with an intracameral air injection, followed by using a 23-gauge needle to puncture the peripheral cornea to drain the pre-descematic fluid. The Descemet’s membrane was completely reattached to the stroma during the follow-up.

**Conclusions:**

Drainage of pre-descematic fluid combined with intracameral air tamponading was used as an alternative surgical option for the management of this severe case of DMD.

## Background

Descemet’s membrane detachment (DMD), a serious complication leading to irreversible corneal decompensation, has been reported following a wide variety of intraocular surgical procedures. Though improvements in phacoemulsification technology have made it possible to perform cataract surgery through microincision and to achieve better postoperative outcomes, a higher incidence of DMD and endothelial gap has been reported [1]. DMD is still one of the most serious complications of modern cataract surgery. The incidence of clinically significant DMD after phacoemulsification varies between 0.044 and 0.5% in phacoemulsification [2, 3].

Different causes as well as a variable course of disease are characteristics of DMD. Possible causal factors include shallow chambers, complicated or repeated operations, the suboptimal quality of surgical equipment, phacoemulsification of hard nuclear cataracts, an inadvertent injection of saline or viscoelastic material in the space between the stroma and Descemet’s membrane, genetically related weak adhesions between the stroma and Descemet’s membrane, et al. [2, 4–6]. Though rare cases of spontaneous reattachment have been reported [7], surgical intervention to promote attachment remains the preferred approach for most patients. Early treatment is essential to achieve visual rehabilitation and to prevent the wrinkling fibrosis and shrinkage of the Descemet’s membrane that can occur over time and result in poor visual outcomes.

Here we present an alternative technique for the treatment of DMD in a patient who underwent a repeat descemetopexy after a failed primary procedure. We also review the literature on current strategies for DMD. To the best of our knowledge, using this surgical approach for the management of DMD after phacoemulsification has not been previously reported.

## Case presentation

**Fig. 1:**
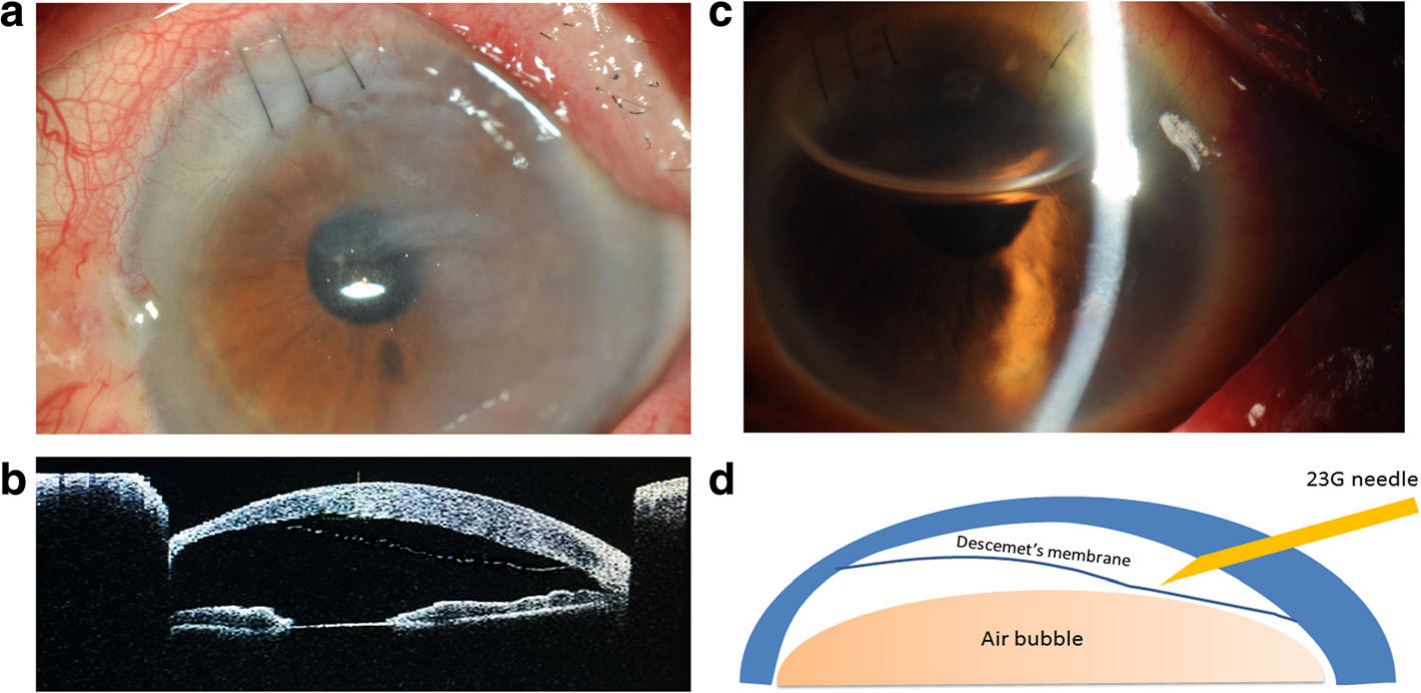
**a** Slit-lamp biomicroscopy of the left eye before treatment. **b** AS-OCT pictures of the left eye with DMD. **c** Postoperative photograph of the left eye with air bubble in the anterior chamber. **d** A schematic illustration of the technique

All procedures conformed to the Declaration of Helsinki, and written informed consent was acquired from the participant. The patient was a 74-year-old woman who underwent clear corneal 3.0-mm incision phacoemulsification surgery on her left eye in the local hospital. One day post-surgery, massive corneal oedema was noticed. DMD was then diagnosed and treated with intracameral air injection. Later, however, the Descemet’s membrane was still detached. The patient was referred to our clinic 1 week postoperatively. Ophthalmologic examination revealed that the best corrected visual acuity (BCVA) was 20/200 in the left eye. Extensive corneal oedema, more temporally with involvement of the visual axis, was observed with slit lamp. Corneal tunnel and puncture incision were sutured with 10–0 nylon sutures. There was detachment of Descemet’s membrane from 11 o’clock to 6 o’clock clockwise with the pupillary axis and peripheral corneal area involved (Fig. 1a). Marginal corneal degeneration was also observed in the superior cornea. An intraocular lens was found stable placed in the posterior chamber. Intraocular pressures (IOP) were 11.0 mmHg in the right eye and 10.5 mmHg in the left eye.

Anterior-segment optical coherence tomography (AS-OCT, Visante, Carl Zeiss Meditec, Dublin, CA) was performed and carefully evaluated for the presence of stromal clefts. A separation of the Descemet’s membrane from the corneal stroma was found by AS-OCT, with the highest point of detachment being at the infratemporal (Fig. 1b). The highest distance of the detached Descemet’s membrane from the posterior stroma was 1.33 mm, measured by AS-OCT with a pattern of linear edge. AS-OCT also revealed significant peripheral corneal thinning, especially in the superior cornea. DMD and cornea arcus senilis were then diagnosed. Similar cornea arcus senilis with peripheral corneal thinning on the right eye was observed with the slit lamp. No keratic precipitates or signs of corneal dystrophy were found.

Given the recurrent nature of the DMD, repeat injection of air bubbles was considered to have less probability of success since insufficient air volume or pre-descematic space fluid might account for the failure of the first attempt to reposition the detached Descemet’s membrane. The possibility of injecting a long-standing gas such as perfluoropropane was discussed but was also considered to be unsafe due to the risk of rupturing the corneal tunnel on the marginal degeneration area. If rupture occurred, repeated suture might be very difficult, and further treatment, such as cornea transplantation, might need to be applied. Pupillary block was also a concern. An alternative technique of draining the pre-descematic fluid and repositioning the detached Descemet’s membrane was proposed. The patient was informed about the procedure and gave informed consent according to the institutional guidelines.

After the eye and periocular area were cleaned with 5% povidone iodine, an anterior chamber paracentesis was performed with a 23-gauge needle at 9 o’clock of the limbus, where the Descemet’s membrane remained in contact. After expressing out some aqueous humor by gently depressing the posterior lip of the paracentesis, the anterior chamber was filled with air with a washing pinhead mounted on a 5 ml syringe. Subsequently, another paracentesis was made with a 23-gauge needle at 5 o’clock of the peripheral cornea as a venting incision, which was the highest point of the detached Descemet’s membrane, using the AS-OCT results as a guide to avoid the visual axial (Fig. 1d). The needle stopped as soon as it penetrated the corneal stroma. Sterilized air was again injected into the anterior chamber through the initial incision. The pre-descematic fluid was noted flowed out through the venting incision. Thirty minutes after surgery, the air was partially removed to prevent pupillary block during the postoperative period. The paracentesis wound was left sutureless. Dexamethasone 2 mg in 0.5 ml was injected subconjunctively at the end of the procedure. Postoperatively, the patient was asked to maintain the supine position for the first 24 hours after the procedure. Topical antibiotics and topical corticosteroids were administered.

One day later, the oedema was lightened, and the cornea had regained much of its clarity. The patient was then suggested to lean to right lateral position for further press of lateral DMD along with the absorption of the air in anterior chamber. Three days after the descemetopexy, the patient’s Descemet’s membrane was completely reattached to the stroma (Fig. 1c) and her BCVA had improved to 20/60. IOP was recorded as less than 21 mmHg during the follow-up. The patient was then discharged and followed up 1 month later in her local hospital. No re-detachment event was reported.

## Discussion and conclusions

The management of Descemet’s detachment depends on various factors such as the location and area of the detachment, the degree of anteroposterior separation from the posterior stroma, and the duration of watchful observation [8]. Due to the unknown course of the disease, exact timing and nature of surgical intervention has not yet been fully determined. There is no gold standard of treatment for DMD.

DMD was first classified as planar and nonplanar (1 mm separation from posterior stroma) in 1977 [9]. In rare cases, the use of topical corticosteroids and hyperosmotics can result in reattachment and resolution of corneal oedema in large persistent DMD after cataract surgery without further surgical intervention [10–13]. Although there have been reports of spontaneous resolution of DMD, the failure rate has been high [7], and the mean time to resolution is also prolonged [14]. Medical treatment alone may not be sufficient, especially in cases of nonplanar DMD.

Surgical repair aims to reapproximate the Descemet’s membrane against the stroma using a tamponading agent until it adheres [8]. Descemetopexy, anterior chamber injection of gas to reposition the detached Descemet’s membrane, is now well accepted for the management of post-cataract surgery DMD due to its ease of execution and subsequent good outcomes [3, 14]. The success rates with intracameral injections have been reported to be 90–95% [15–17]. Tamponading agents successfully used for this purpose include 100% air, sulphur hexafluoride (15–20% SF6), and perfluoropropane (12–14% C3F8). Air is usually preferred for many reasons, including a shorter time of absorption, lower cost, and less risk of endothelial toxicity or pupillary block than with other long-standing gases [8, 17]. SF6 and C3F8 with their longer resorption time were selected for cases of failing reattachment with air or of detachment for a prolonged period of time. Repeated injections with air or other gases are sometimes required to reposit the DMD [2, 17]. Tamponading with viscoelastic agents has also been reported as being successful [18, 19]. Due to the high risk of increasing the IOP and the need for constant monitoring, this method has been used only in cases with recalcitrant DMD despite simple pneumodescemetopexy.

One study reported the use of Nd:YAG laser to treat a fluid-filled DMD by draining the fluid into the anterior chamber via openings made in Descemet’s membrane in a patient 19 months after cataract surgery [20]. However, this procedure could not be performed on our patient due to the corneal opacity. Transcorneal suturing of the detached Descemet’s membrane has also been reported [21]. In inferior detachments, the application of sutures has been reported with favourable results [22]. Combined intracameral gas and transcorneal suturing were reported to be effective in the repair of DMD that failed to reattach with air alone [23]. However, this technique is more invasive and full-thickness suture could cause stretch lines, making it a less desirable option.

If all the mentioned interventions fail, keratoplasty—either selective endothelial keratoplasty or conventional penetrating keratoplasty—may be needed to restore vision [3]. Keratoplasty has its own inherent limitations, such as nonavailability of corneal tissue, requirement of good postoperative care and regular follow-up, and risk of rejection and infection [24]. Thus repeat descemetopexy for DMD after cataract surgery is worthwhile before initiating a complex surgical procedure.

Why had this patient developed serious DMD after phacoemulsification? A logical explanation for this unusual presentation is that the surgeon was inexperienced. Marginal corneal degeneration might also have been involved in the progression of DMD. According to the AS-OCT, the Descemet’s membrane detached from the temporal cornea without rupture or rolled scroll, which suggests that DMD was likely caused by the inflow of saline or viscoelastic material at the incision site between the stroma and Descemet’s membrane, which might not have been noticed during surgery. This patient had a massive DMD, involving almost 60% of the cornea, and the first descemetopexy with air tamponade failed. Intracameral injection of C3F8 or SF6 was not a good option due to the high probability of an increase in IOP, which could have caused splitting of the primary sutured corneal tunnel or pupillary block. Considering the various patient-related factors required for the long-term survival of a corneal graft, the best outcome would be obtained if the patient’s own Descemet’s membrane could be repositioned in its anatomic place.

Corneal venting incisions have been successfully used to drain the fluid in the pre-descematic area in operated cases of Descemet’s stripping endothelial keratoplasty, acute corneal hydrops with tears in the Descemet’s membrane and multiple intrastromal clefts [25, 26]. With this patient we applied a similar procedure to reattach the detached Descemet’s membrane and hasten the resolution of corneal oedema by creating a venting incision to drain out the fluid from the pre-descematic space. The key points of this operation include carefully selecting the peripheral incision site of air injection to avoid further DMD, choosing the external stab incision at the highest point of DMD for drainage of pre-descematic liquid and avoiding the area of visual axis. A complete air fill in the anterior chamber for at least 10 min gives enough time to drain the pre-descematic fluid through the venting incisions and ensures Descemet’s membrane reattachment on a permanent basis [26]. Subsequent decompression of air is performed later to prevent postoperative pupillary block. Air tamponade with proper head position provides enough pressure after the operation.

Prognosis depends on prompt recognition and early treatment of DMD. AS-OCT is effective for early diagnosing DMD, guiding subsequent treatment, and monitoring progress of DMD in eyes with dense corneal edema [5]. This alternative technique might be a minimally invasive technique that can be performed to eventually reposition the DMD without tamponading with long-standing gases in this rare case. However, corneal scarring and astigmatism might be an undesirable complication in this case. These risks can be lowered by carefully choosing the venting position at the highest location of the detached Descemet’s membrane along with avoiding the visual axial.

In summary, drainage of pre-descematic fluid combined with intracameral air tamponading was used as an alternative surgical option for the management of this severe case of DMD. It would be better for the surgeon to pay particular attention to the DMD while performing phacoemulsification, especially in patients with peripheral cornea disorder.

## Abbreviations

AS-OCT: Anterior-segment optical coherence tomography
DMD: Descemet’s membrane detachment
IOP: Intraocular pressure
